# Human adenoviruses in children with gastroenteritis: a systematic review and meta-analysis

**DOI:** 10.1186/s12879-024-09386-x

**Published:** 2024-05-09

**Authors:** Pegah Khales, Mohammad Hossein Razizadeh, Saied Ghorbani, Afagh Moattari, Jamal Sarvari, Hassan Saadati, Shirin Sayyahfar, Zahra Salavatiha, Morteza Haghighi Hasanabad, Vahdat Poortahmasebi, Ahmad Tavakoli

**Affiliations:** 1 Department of Bacteriology and Virology, School of Medicine, Shiraz University of Medical Sciences, Shiraz, Iran; 2 Department of Virology, School of Medicine, Iran University of Medical Sciences, Tehran, Iran; 3 Antimicrobial Resistance Research Center, Institute of Immunology and Infectious Diseases, Iran University of Medical Sciences, Tehran, Iran; 4 Department of Epidemiology and Biostatistics, School of Health, North Khorasan University of Medical Sciences, Bojnurd, Iran; 5 Research Center of Pediatric Infectious Diseases, Institute of Immunology and Infectious Diseases, Iran University of Medical Sciences, Tehran, Iran; 6 Department of Bacteriology and Virology, Faculty of Medicine, Tabriz University of Medical Sciences, Tabriz, Iran; 7 Infectious and Tropical Diseases Research Center, Tabriz University of Medical Sciences, Tabriz, Iran

**Keywords:** Gastroenteritis, Human adenoviruses, Pediatrics, Epidemiology, Children

## Abstract

**Purpose:**

Human adenoviruses (HAdVs) have always been suggested as one of the main causes of gastroenteritis in children. However, no comprehensive report on the global epidemiology of these viruses in pediatric gastroenteritis is available.

**Methods:**

A systematic search was conducted to obtain published papers from 2003 to 2023 in three main databases PubMed, Scopus, and Web of Science.

**Results:**

The estimated global pooled prevalence of HAdV infection in children with gastroenteritis was 10% (95% CI: 9-11%), with a growing trend after 2010. The highest prevalence was observed in Africa (20%, 95% CI: 14–26%). The prevalence was higher in inpatients (11%; 95% CI: 8-13%) and patients aged 5 years old and younger (9%; 95% CI: 7-10%). However, no significant difference was observed between male and female patients (*P* = 0.63). The most prevalent species was found to be the species F (57%; 95% CI: 41-72%). The most common HAdVs observed in children with gastroenteritis were types 40/41, 38, and 2. Analysis of case-control studies showed an association between HAdV and gastroenteritis in children (OR: 2.28, 95% CI; 1.51–3.44).

**Conclusion:**

This study provided valuable insights into the importance of HAdVs in children with gastroenteritis, especially in hospitalized and younger children. The results can be used in future preventive measurements and the development of effective vaccines.

**Supplementary Information:**

The online version contains supplementary material available at 10.1186/s12879-024-09386-x.

## Introduction

Acute gastroenteritis is a serious threat to health that affects individuals of any age. It is especially serious for the very young, such as newborns and young children [1, 2]. Because of their underdeveloped immunity, children are more susceptible to diarrheal illnesses. Different enteric pathogens, including bacteria, viruses, protozoa, helminths, and fungi, can cause diarrhea. These pathogens are typically transmitted by ingesting contaminated food, water, or things infected with feces [3]. Previous studies have shown that the virus is the most common cause of acute gastroenteritis in individuals younger than 18 years of age [4, 5]. The most common causes of acute gastroenteritis in children are rotavirus, norovirus (NoV), human adenovirus (HAdV), and human astrovirus (HAstV) [6, 7].

HAdV is a member of the *Adenoviridae* family and the *Mastadenovirus* genus. HAdV is a non-enveloped, medium-sized virus (70–100 nm) with an icosahedral nucleocapsid that contains a 34–45 kbp double-stranded linear DNA genome [8, 9]. HAdVs have been divided into seven species A to G based on pathogenicity and genetic features, with 115 distinct HAdVs genotypes being identified [10]. Based on the percentage of guanine plus cytosine in their DNA and other biochemical and biophysical criteria which are classified into 7 species (A-G). The word serotype is used to point to types up to 51 while newer types, which were differentiated by novel sequences or recombinant phylogeny in genes coding for major capsid proteins are known as genotype. Species G is composed of one type (type 52) and is extremely rare while other species are found in patients with various diseases including gastroenteritis, conjunctivitis, respiratory infections, and to a lesser extent in intussusception in infants, hemorrhagic cystitis, meningoencephalitis, myocarditis, and hepatitis [11]. HAdV infection, a highly infectious disease, can infect a range of organs, including upper and lower respiratory tracts, gastrointestinal tract, urinary tract, eye, and other systems [11]. . Tissue tropisms vary by species. It has been determined that the primary cause of acute gastroenteritis among the seven species is the HAdV F species, also known as enteric HAdV, which contains the HAdV-F40 and HAdV-F41 genotypes [12–15]. Enteric species F (genotypes F40/41) strains were predicted using a mathematical model to be the third most common agent responsible for mortality in diarrheal children under the age of five, after rotavirus and Shigella [16]. Furthermore, stool samples from patients with acute gastroenteritis have regularly revealed the presence of several other non-enteric HAdV species (HAdV A-E and G species), including HAdV A, B, C, and D [12, 13, 15, 17–20].

While there are many reports from different parts of the world, there is a gap of knowledge in understanding the epidemiology and association of HAdVs and pediatric gastroenteritis. This study aims to fulfill this gap by comprehensively analysis various factors including age group, gender, geographical teacher, clinical setting, diagnostics methods, species, and genotypes in pediatrics gastroenteritis for the first time to provide valuable insights into the current status of HAdVs in children with gastroenteritis.

## Methods

The Preferred Reporting Items for Systematic Reviews and Meta-Analyses (PRISMA) guideline served as the foundation for this systematic review and meta-analysis approach [21].

### Search strategy

To discover relevant papers, a systematic literature search was undertaken utilizing three electronic databases including PubMed, Scopus, and Web of Science. The literature search was restricted to the period between inception to June 24, 2023. Table S1 provides information about the search terms for each database. We manually searched the reference lists of pertinent articles to find further research that met the eligibility criteria. For data management, the systematic literature search was loaded into EndNote software version X8 (Thomson Reuters, California, USA).

### Selection criteria

Studies were considered qualified if they reported: (1) case-control and cross-sectional studies providing data related to the prevalence of enteric and non-enteric HAdVs among children less than 18 years with gastroenteritis published in the English language in peer-reviewed journals; (2) the prevalence of HAdV genome in stool samples and rectal swabs; (3) studies detecting HAdV genome by polymerase chain reaction (PCR)-based methods; (4) studies detecting the prevalence of HAdV among inpatients and outpatients; (5) original articles and short communications with sufficient data. Studies that met any of the following criteria were excluded: (1) the prevalence of HAdV infection among adults patients with gastroenteritis; (2) the prevalence of HAdV infection among children presenting gastroenteritis with underlying conditions such as transplant recipients, HIV, immunocompromised status, and cancers; (3) the incidence of HAdV infection among children with gastroenteritis; (4) samples other than stool such as oral swabs, serum, cerebrospinal fluid, and conjunctival swabs; (5) detection of HAdV by assays othe than PCR-based methods such as antigen detection assays, immunochromatography, Loop-mediated isothermal amplification, next-generation sequencing-based viral metagenomics, microarray, latex agglutination, electronmicroscopy, DNA restriction enzyme analysis, enzyme immunoassay, culture techniques, immunoelectron microscopy, and nucleic acid hybridization; (6) seroprevalence of HAdV antibodies; (7) studies included patients with non-gastroenteritis symptoms such as respiratory symptoms, acute severe hepatitis, and asymptomatic; (8) letters, case series, notes, review articles, case reports, posters, and conference abstracts; (9) articles published in languages other than English.

### Data extraction and quality assessment

Two reviewers separately examined the titles and abstracts of all identified papers, and studies that were unrelated to the study topic were eliminated. The reviewers got full texts of the selected papers and further analyzed them, and those that did not meet the inclusion criteria were excluded. Finally, any differences among reviewers were settled by consulting with a third reviewer. Utilizing a modified checklist based on strengthening the reporting of observational studies in epidemiology (STROBE), a quality assessment of the retrieved studies was carried out [22, 23]. The checklist consisted of 12 questions that addressed various methodological approaches. Studies that received a validity score of at least 8 out of a maximum of 12 were considered eligible for the main meta-analysis. One reviewer extracted the data listed below from each eligible article: first author’s last name, year of publication, year of sampling, study location, study design, sample size, age ranges of patients, age groups of patients, the gender of patients, number of HAdV-positive cases, HAdV detection methods, types of patient care, species, and genotype of HAdV. The retrieved data were entered into a pre-designed Excel spreadsheet (Microsoft Corporation, Redmond, Washington, USA).

### Statistical analysis

We pooled the HAdV infection in children suffering from gastroenteritis using the metaprop package [24]. We applied the random-effects meta-analysis framework and subgroup analysis was conducted based on region, gender, age, detection method, sampling time, types of patient care, and genotype of HAdV. We also conducted meta-analyses of risk estimates for gastroenteritis and exposure to HAdV, and we reported pooled estimates of odds ratio (OR) and 95% CIs. DerSimonian and Laird method was used to compute the pooled estimate of OR with confidence interval (95% CI) using random models. Statistical heterogeneity between studies was evaluated with Cochran’s Q test and quantified by I^2^ statistic [25]. We investigated the presence and the effect of publication bias using a combination of the visual inspection of funnel plots that were constructed, plotting the logarithmically transformed ORs against the standard error of the associated log (OR) and Begg’s test and Egger’s test. All statistical tests were two-tailed and the significance level was considered less than 0.05 for all, except heterogeneity test that were set at less than 0.1, and statistical analyses were performed using Stata 14.1 (Stata Corp, College Station, TX, USA).

## Results

### Literature search

**Fig. 1:**
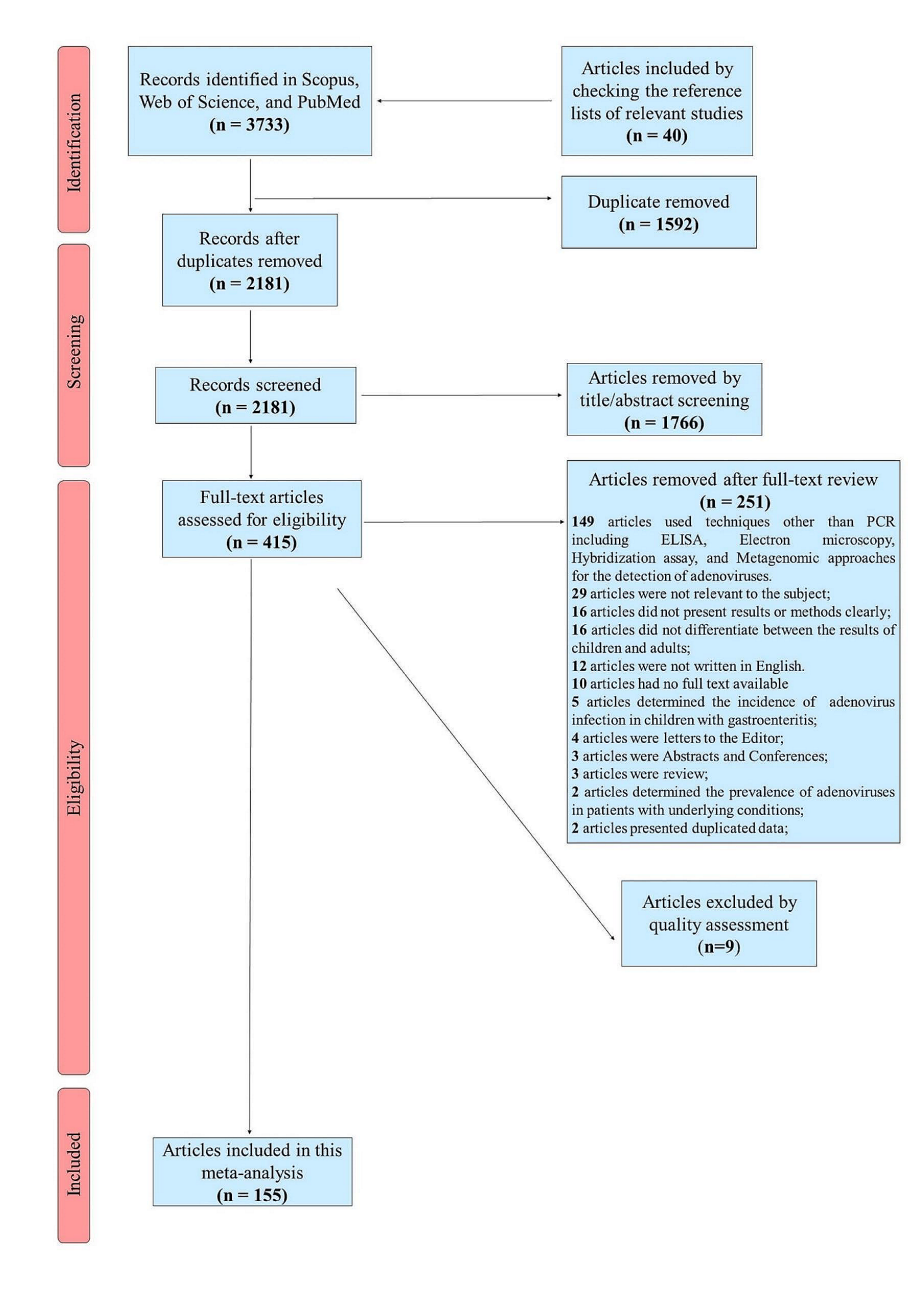
Flowchart presenting the steps of literature search and selection

During the initial search, 3733 papers were identified, and 40 further papers were discovered by manually examining the reference lists of pertinent research. A total of 1592 duplicate papers were initially removed, and 1766 additional papers were removed after a manual check of titles and abstracts. After a thorough evaluation of the full text of the remaining 415 papers to determine their eligibility for the meta-analysis, 251 of them were removed. According to the modified STROBE checklist, 155 publications were deemed to be of good quality (scoring of 8 or higher), with 9 papers were failed to get a score of 8. Finally, this systematic review and meta-analysis contained 155 papers. An overview of the selection of relevant studies is depicted in Fig. 1.

### Study characteristics

Out of the 155 studies considered, 134 were cross-sectional and 21 were case-control in design. The articles’ publication dates varied from 2003 to 2023. The largest research involved 85,001 gastroenteritis cases [26], while the smallest contained 24 cases [27]. Out of the 155 papers included in this meta-analysis, 19 research examined the gender distribution of HAdV infection, and 80 studies looked into the genotype distribution of HAdVs. Specific primers for the detection of HAdVs group F (types 40 and 41) and universal primers identifying all types of HAdVs have been used in 37 and 118 studies, respectively. The majority of study populations (*n* = 103,815) were children under 5 years of age and 12,982 were children between the ages of 6 and 18 years old. The majority of studies (*n* = 29) were conducted in China, followed by Brazil (*n* = 12), India (*n* = 11), and Japan (*n* = 10). Regarding the continent, 84 were conducted in Asia, 26 in South America, 18 in Europe, 16 in North America, 9 in Africa, and 2 in Oceania. The characteristics of included studies in this systematic review and meta-analysis are summarized in Table 1.

**Table 1:** Characteristics of studies included in the systematic review and meta-analysis

Author (Ref)	Publication Year	Location	Study design	Age Range	Number of cases	No. Positive in cases	Number of controls	No. Positive in controls
Oh [28]	2003	Germany	Cross-Sectional	29 days to 15.5 years	217	31		
Phan [29]	2004	Japan	Cross-Sectional	2 months to 14 years	236	9		
Yan [30]	2004	China	Cross-Sectional	Under 7 years	207	12		
Akihara [31]	2005	Japan	Case-Control	1 month to 2 years	88	11	833	96
Logan [32]	2006	Ireland	Cross-Sectional	Under 18 years	220	11		
Phan [33]	2006	Japan	Cross-Sectional	5 months to 8 years	125	1		
Reither [34]	2007	Ghana	Case-Control	Under 12 years	243	67	124	39
Chen [35]	2007	Taiwan	Cross-Sectional	3 months to 18 years	257	51		
Fabiana [36]	2007	Italy	Cross-Sectional	2 months to 12 years	313	29		
Nguyen [37]	2007	Vietnam	Cross-Sectional	37 days to 9 years	1010	32		
Shimizu [38]	2007	Japan	Cross-Sectional	3 months to 14 years	337	27		
Gomara [39]	2008	UK	Cross-Sectional	Under 6 years	685	66		
Jin [40]	2008	China	Cross-Sectional	Under 5 years	1110	85		
Silva [41]	2008	Ghana	Cross-Sectional	Under 11 years	367	73		
Verma [42]	2008	India	Cross-Sectional	Under 5 years	439	34		
Dey [43]	2009	Japan	Cross-Sectional	Under 10 years	628	28		
Dey [44]	2009	Bangladesh	Cross-Sectional	2 months to 3.2 years	917	17		
Jin [45]	2009	China	Cross-Sectional	Under 5 years	544	18		
Kittigul [46]	2009	Thailand	Cross-Sectional	Under 15 years	131	4		
Li [47]	2009	Hong Kong	Cross-Sectional	Under 18 years	209	7		
Nakanishi [48]	2009	Japan	Cross-Sectional	Under 14 years	877	33		
Podkolzin [49]	2009	Russia	Cross-Sectional	Under 14 years	3208	119		
Sdiri-Loulizi [50]	2009	Tunisia	Cross-Sectional	Under 12 years	788	18		
Cunliffe [51]	2010	UK	Cross-Sectional	Under 16 years	576	83		
Rasanen [52]	2010	Finland	Cross-Sectional	Under 15 years	50	5		
Zhang [53]	2011	China	Case-Control	Under 5 years	201	10	53	5
Khamrin [54]	2011	Japan	Cross-Sectional	Under 5 years	235	8		
Rimoldi [55]	2011	Italy	Cross-Sectional	Under 18 years	273	4		
Braun [27]	2012	USA	Case-Control	Under 2 years	24	11	78	43
Chaimongkol [56]	2012	Thailand	Cross-Sectional	Under 5 years	160	3		
Grant [57]	2012	USA	Cross-Sectional	Under 9 months	247	3		
Lee [58]	2012	South Korea	Cross-Sectional	Under 18 years	2064	113		
Ouyang [59]	2012	China	Cross-Sectional	Under 5 years	766	135		
Rezaei [60]	2012	Iran	Cross-Sectional	Under 5 years	100	8		
Seo [61]	2012	South Korea	Cross-Sectional	Under 10 years	310	48		
Chhabra [62]	2013	USA	Case-Control	Under 5 years	782	93	499	9
Al-Thani [63]	2013	Qatar	Cross-Sectional	Under 10 years	121	11		
Chen [64]	2013	China	Cross-Sectional	Under 5 years	811	22		
Chen [65]	2013	Taiwan	Cross-Sectional	Under 18 years	755	69		
Dey [66]	2013	Japan	Cross-Sectional	Under 15 years	7185	565		
Ren [67]	2013	China	Cross-Sectional	Under 5 years	477	30		
So [68]	2013	South Korea	Cross-Sectional	1 month to 11 years	186	0		
Zhu [69]	2013	China	Cross-Sectional	Under 3 years	749	6		
Kabayiza [70]	2014	Rwanda	Case-Control	Under 5 years	544	216	162	68
Chhabra [71]	2014	Soviet Union	Cross-Sectional	Under 5 years	495	20		
Kabayiza [72]	2014	Rwanda	Cross-Sectional	Under 5 years	880	216		
Liu [73]	2014	China	Cross-Sectional	Under 6 years	2233	219		
Mitui [74]	2014	Turkey and Bangladesh	Cross-Sectional	Under 5 years	288	168		
Raboni [75]	2014	Brazil	Cross-Sectional	Under 5 years	225	45		
Soli [76]	2014	New Guinea	Cross-Sectional	Under 5 years	199	23		
Amaral [77]	2015	Brazil	Cross-Sectional	Under 5 years	591	12		
Chen [78]	2015	Taiwan	Cross-Sectional	Under 5 years	2810	105		
Khoshdel [79]	2015	Iran	Cross-Sectional	Under 5 years	100	22		
La Rosa [19]	2015	Albania	Cross-Sectional	2 months to 7 years	142	33		
Lekana-Douki [80]	2015	Gabon	Cross-Sectional	Under 5 years	317	62		
Liu [81]	2015	China	Cross-Sectional	Under 5 years	2171	150		
Lu [82]	2015	China	Cross-Sectional	Under 5 years	436	31		
Mladenova [83]	2015	Bulgaria	Cross-Sectional	Under 3 years	115	11		
Osborne [84]	2015	USA	Cross-Sectional	Under 18 years	941	95		
Patil [85]	2015	India	Cross-Sectional	Under 9 years	950	12		
Thongprachum [86]	2015	Japan	Cross-Sectional	Under 15 years	2381	134		
Yu [87]	2015	China	Cross-Sectional	Under 5 years	18,266	879		
Zhang [88]	2015	China	Cross-Sectional	Under 14 years	1128	76		
Li [89]	2016	China	Case-Control	Under 5 years	461	50	461	12
Ouédraogo [90]	2016	Burkina Faso	Case-Control	Under 5 years	263	82	50	25
Steyer [91]	2016	Slovenia	Case-Control	Under 6 years	297	22	88	0
Brown [92]	2016	UK	Cross-Sectional	Under 18 years	1393	146		
Dashti [93]	2016	Iran	Cross-Sectional	Under 5 years	2682	132		
Jin [94]	2016	South Korea	Cross-Sectional	1 month to 16 years	345	26		
Liu [95]	2016	China	Cross-Sectional	Under 5 years	3147	324		
Nakamura [96]	2016	Japan	Cross-Sectional	Under 15 years	1796	88		
Reis [97]	2016	Brazil	Cross-Sectional	Under 12 years	377	47		
Shen [98]	2016	China	Cross-Sectional	Under 18 years	137	3		
Colak [99]	2017	Turkey	Cross-Sectional	Under 5 years	180	25		
Cornejo-Tapia [100]	2017	Peru	Cross-Sectional	Under 5 years	117	17		
Costa [101]	2017	Brazil	Cross-Sectional	Under 2 years	172	74		
Hawash [102]	2017	Saudi Arabia	Cross-Sectional	Under 18 years	76	5		
Kim [103]	2017	South Korea	Cross-Sectional	Under 16 years	415	56		
Lu [104]	2017	China	Cross-Sectional	Under 5 years	674	32		
Stockmann [105]	2017	USA	Cross-Sectional	Under 18 years	1089	71		
Zaki [106]	2017	Egypt	Cross-Sectional	Under 5 years	100	20		
Qiu [107]	2018	China	Case-Control	Under 18 years	273	79	361	26
Adam [108]	2018	Sudan	Cross-Sectional	Under 5 years	437	7		
Alcala [109]	2018	Venezuela	Cross-Sectional	Under 5 years	227	26		
Biscaro [110]	2018	Italy	Cross-Sectional	2 months to 15 years	510	35		
Primo [111]	2018	Brazil	Cross-Sectional	Under 10 years	2009	107		
Yu [112]	2018	Taiwan	Cross-Sectional	Under 5 years	837	13		
Hassan [113]	2019	USA	Case-Control	Under 2 years	330	75	272	44
Iturriza-Gomara [114]	2019	Malawi	Case-Control	Under 5 years	684	199	527	14
Lima [115]	2019	Brazil	Case-Control	2 months to 3 years	588	19	573	5
Shen [116]	2019	China	Case-Control	Under 18 years	273	24	361	16
Tilmanne [117]	2019	Belgium	Case-Control	Under 16 years	178	12	165	5
Arashkia [118]	2019	Iran	Cross-Sectional	Under 5 years	376	16		
Arowolo [119]	2019	Nigeria	Cross-Sectional	Under 5 years	175	9		
Elmahdy [120]	2019	Egypt	Cross-Sectional	Under 5 years	60	17		
Gaensbauer [121]	2019	Guatemala	Cross-Sectional	6 to 35 months	316	41		
Gelaw [13]	2019	Ethiopia	Cross-Sectional	Under 5 years	450	144		
Goldar [122]	2019	India	Cross-Sectional	6 months to 5 years	80	27		
Harb [123]	2019	Iraq	Cross-Sectional	Under 5 years	155	53		
Kumthip [12]	2019	Thailand	Cross-Sectional	Under 5 years	2312	165		
Portal [124]	2019	Brazil	Cross-Sectional	Under 9 years	219	110		
Pratte-Santos [125]	2019	Brazil	Cross-Sectional	Under 12 years	134	81		
Tatte [126]	2019	India	Cross-Sectional	Under 5 years	185	5		
Theamboonlers [127]	2019	Thailand	Cross-Sectional	Under 15 years	442	87		
Farfan-Garcia [128]	2020	Colombia	Case-Control	Under 5 years	431	14	430	1
Pabbaraju [129]	2020	Canada	Case-Control	Under 18 years	3347	629	1355	97
Dey [130]	2020	Bangladesh	Cross-Sectional	Under 15 years	574	24		
Kim [131]	2020	South Korea	Cross-Sectional	Under 5 years	740	7		
Lambisia [132]	2020	Kenya	Cross-Sectional	Under 5 years	984	120		
Mohammadi [133]	2020	Iran	Cross-Sectional	Under 5 years	103	3		
Mousavi Nasab [134]	2020	Iran	Cross-Sectional	Under 5 years	120	6		
Romo-Saenz [135]	2020	Mexico	Cross-Sectional	Under 5 years	57	8		
Sharif [136]	2020	Bangladesh	Cross-Sectional	Under 15 years	387	22		
Zhu [137]	2020	China	Cross-Sectional	Under 5 years	1220	37		
Alsuwaidi [138]	2021	UAE	Case-Control	Under 5 years	203	35	73	2
Harrison [139]	2021	USA	Case-Control	Under 11 years	660	51	624	9
Huang [140]	2021	China	Case-Control	Under 5 years	383	21	327	13
Mero [141]	2021	Guinea-Bissau	Case-Control	Under 5 years	228	40	201	32
Abdel-Rahman [142]	2021	Qatar	Cross-Sectional	3 months and 14 years	901	59		
Barsoum [143]	2021	Ireland	Cross-Sectional	Under 3 years	150	19		
Chandra [144]	2021	India	Cross-Sectional	Under 5 years	3882	351		
Chang [145]	2021	China	Cross-Sectional	Under 18 years	2692	193		
De Francesco [146]	2021	Italy	Cross-Sectional	Under 18 years	476	34		
Gopalkrishna [147]	2021	India	Cross-Sectional	Under 5 years	308	25		
Huang [14]	2021	China	Cross-Sectional	Under 5 years	656	49		
Lu [148]	2021	China	Cross-Sectional	Under 5 years	804	28		
Ndjangangoye [149]	2021	Gabon	Cross Sectional	Under 15 years	66	54		
Olivares [150]	2021	Brazil	Cross-Sectional	Under 5 years	458	139		
Rossouw [151]	2021	South Africa	Cross-Sectional	Under 5 years	221	15		
Souza [152]	2021	Brazil	Cross-Sectional	Under 18 years	1992	166		
Souza [153]	2021	Brazil	Cross-Sectional	Under 14 years	3419	171		
Wang [26]	2021	China	Cross-Sectional	Under 18 years	85,001	2284		
Abbasi [154]	2022	Iran	Cross-Sectional	Under 7 years	173	4		
Allayeh [155]	2022	Egypt	Cross-Sectional	Under 5 years	447	35		
Al-Nasrawy [156]	2022	Iraq	Cross-Sectional	Under 3 years	450	150		
Colito [157]	2022	Cape Verde	Cross-Sectional	Under 12 years	105	7		
do Nascimento [158]	2022	Brazil	Cross-Sectional	Under 18 years	1012	227		
Dong [159]	2022	China	Cross-Sectional	Under 5 years	897	106		
Gelaw [160]	2022	Ethiopia	Cross-Sectional	Under 5 years	38	7		
Jo [161]	2022	South Korea	Cross-Sectional	Under 9 years	184	1		
Li [162]	2022	China	Cross-Sectional	Under 14 years	160	15		
Mihala [163]	2022	Australia	Cross-Sectional	Under 2 years	11,111	2171		
Mitra [164]	2022	India	Cross-Sectional	Under 5 years	3157	276		
Othma [165]	2022	Egypt	Cross-Sectional	Under 5 years	50	3		
Shams [166]	2022	Iran	Cross-Sectional	Under 15 years	130	23		
Tang [20]	2022	China	Cross-Sectional	Under 14 years	1352	60		
Yılmaz [167]	2022	Turkey	Cross-Sectional	Under 18 years	94	13		
Bhat [168]	2023	India	Cross-Sectional	1 month to 18 years	109	0		
Borkakoty [169]	2023	India	Cross-Sectional	Under 5 years	407	187		
Eifan [170]	2023	Saudi Arabia	Cross-Sectional	Under 18 years	97	6		
Hugho [3]	2023	Tanzania	Cross-Sectional	Under 5 years	146	29		
Joshi [16]	2023	India	Cross-Sectional	Under 5 years	1167	61		
Lu [171]	2023	China	Cross-Sectional	Under 15 years	1048	97		
Ndjangangoye [172]	2023	Gabon	Cross Sectional	Under 15 years	284	75		
Potgieter [173]	2023	South Africa	Cross-Sectional	Under 5 years	275	52		

### Prevalence of HAdV infection among children with gastroenteritis

The estimated global pooled prevalence of HAdV infection among 222,267 gastroenteritis-affected children from 51 countries was 10% (95% CI: 9-11%; I²=98.6%; *P* < 0.001). By age, children aged 13 to 24 months had a slightly greater prevalence of HAdV (14%; 95% CI: 9-20%) than children of other ages (*P* = 0.56). The frequency of HAdV infection was similar between males and females (8%; 95% CI: 6-11% vs. 8%; 95% CI: 6-10%, respectively; *P* = 0.63) (Table 2).

**Fig. 2:**
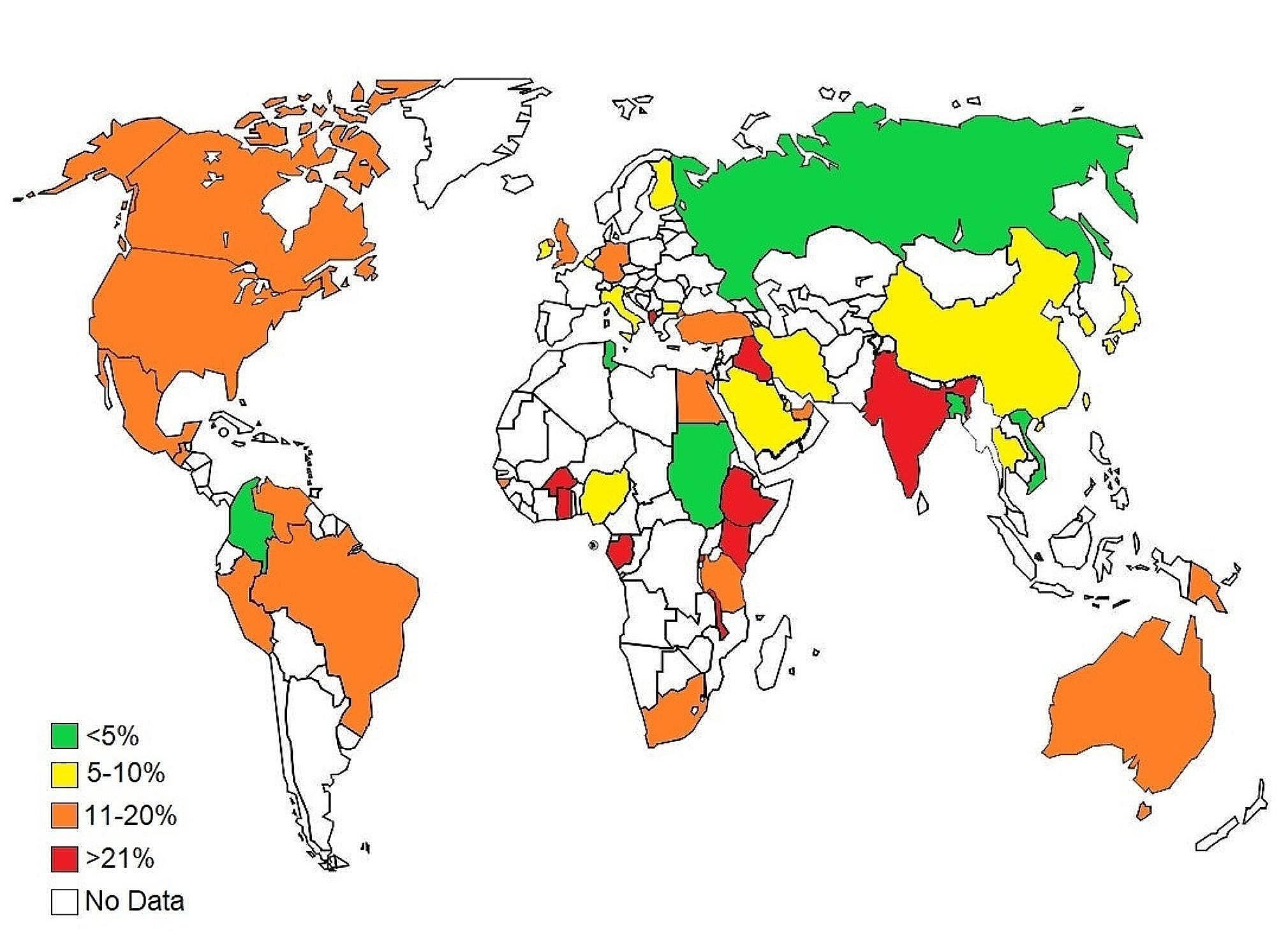
The global map presents the geographical variations in the prevalence of HAdV infection among pediatric patients with gastroenteritis in a period of 11 years (2003–2023)

According to our subgroup analysis, the highest prevalence of HAdV infection was seen in pediatric gastroenteritis patients from Gabon (42%, 95% CI: 16-70%), followed by Iraq (34%, 95% CI: 30-37%), Ethiopia (31%, 95% CI: 27-35%), and Rwanda (30%, 95%CI: 28-33%). Figure 2 depicts the global distribution of HAdV infection among children with gastroenteritis.

With respect to HAdV detection methods, Nested PCR, Multiplex PCR, Real-time PCR, Conventional PCR, and Multiplex Real-time PCR methods were used. The prevalence of HAdV was 23% (95% CI: 12–37%), 5% (95% CI: 4–6%), 15% (95% CI: 12–19%), 9% (95% CI: 8–11%), and 17% (95% CI: 10–26%), when Nested PCR, Multiplex PCR, Real-time PCR, Conventional PCR, and Multiplex Real-time PCR methods were used, respectively (*P* < 0.001). Regarding patient setting, the higher prevalence of HAdV was found in inpatients than in outpatients (9%; 95% CI: 7-10% vs. 7%; 95% CI: 5-8%, respectively); however, the difference was not statistically significant *(P* = 0.09) (Table 2).

A time trend analysis was conducted to assess variations in the prevalence of HAdV infection over time throughout the world. According to this analysis, the prevalence of HAdV was the highest (32%; 95% CI: 26-37%) between the years of 1996 and 2000. Since 2001 until 2010, the number of HAdV-positive cases among pediatric patients with gastroenteritis was dramatically decreased, so that the prevalence was 8% (95% CI: 4-12%) between the years of 2001 and 2005, and 8% (95% CI: 6-9%) between the years of 2006 and 2010. However, the prevalence of HAdV infection was remarkably increased after the year 2010, reaching a peak of 13% (95% CI: 6-21%) during the years of 2021 and 2022 (*P* < 0.001) (Table 2).

Regarding the continent, Africa showed a higher prevalence of HAdV in pediatric patients with gastroenteritis (20%, 95% CI: 14–26%) compared to Oceania (19%, 95% CI: 19–20%), the South America (16%, 95% CI: 10–22%), the North America (12%, 95% CI: 8–18%), Europe (9%, 95% CI: 7–12%), and the Asia (7%, 95% CI: 6–8%) (*P* < 0.001) (Table 2).

**Table 2:** Subgroup analysis of the prevalence of HAdV infection among pediatric patients with gastroenteritis

	Group	Number of studies	Pooled prevalence (%) (95%CI)	Heterogeneity test I^2^%, *p*-value	Differences between subgroups; χ2 test (*p*-value)
**Overall prevalence**	-	155	0.10 (0.09–0.11)	98.63, < 0.001	
**Study design**	Cross-sectional	134	0.10 (0.08–0.11)	98.58, < 0.001	***P*** **= 0.01**
	Case-control	21	0.15 (0.11–0.20)	97.32, < 0.001	
**Method**	Nested PCR	8	0.23 (0.12–0.37)	98.17, < 0.001	***P*** **< 0.001**
	Multiplex PCR	44	0.05 (0.04–0.06)	94.27, < 0.001	
	Real-time PCR	29	0.15 (0.12–0.19)	98.27, < 0.001	
	Conventional PCR	61	0.09 (0.08–0.11)	98.09, < 0.001	
	Multiplex Real-time PCR	13	0.17 (0.10–0.26)	97.74, < 0.001	
**Primer**	Universal	118	0.10 (0.09–0.12)	98.84, < 0.001	*P* = 0.60
	Group F	37	0.10 (0.08–0.12)	95.87, < 0.001	
**Sampling time**	1996–2000	2	0.32 (0.26–0.37)	NA	***P*** **< 0.001**
	2001–2005	16	0.08 (0.04–0.12)	97.74, < 0.001	
	2006–2010	36	0.08 (0.06–0.09)	95.15, < 0.001	
	2011–2015	42	0.12 (0.10–0.15)	98.88, < 0.001	
	2016–2020	51	0.11 (0.08–0.13)	97.21, < 0.001	
	2021–2022	8	0.13 (0.06–0.21)	99.14, < 0.001	
**Continent**	South America	26	0.16 (0.10–0.22)	98.61, < 0.001	***P*** **< 0.001**
	Asia	84	0.07 (0.06–0.08)	97.70, < 0.001	
	Europe	18	0.09 (0.07–0.12)	92.45, < 0.001	
	Africa	9	0.20 (0.14–0.26)	97.93, < 0.001	
	North America	16	0.12 (0.08–0.18)	96.96, < 0.001	
	Oceania	2	0.19 (0.19–0.20)	NA	
**Gender**	Male	19	0.08 (0.06–0.11)	90.64, < 0.001	*P* = 0.63
	Female	19	0.08 (0.06–0.10)	87.95, < 0.001	
**Age (month)**	0–6	18	0.08 (0.05–0.12)	93.26, < 0.001	*P* = 0.56
	7–12	17	0.09 (0.06–0.13)	95.36, < 0.001	
	13–24	19	0.14 (0.09–0.20)	95.31, < 0.001	
	25–36	12	0.11 (0.04–0.19)	90.97, < 0.001	
	37–48	8	0.10 (0.02–0.22)	86.23, < 0.001	
	49–60	8	0.06 (0.00-0.16)	80.80, < 0.001	
**Age (year)**	0–5	38	0.11 (0.08–0.13)	98.49, < 0.001	***P*** **< 0.001**
	6–18	23	0.04 (0.02–0.05)	83.87, < 0.001	
**Patient type**	Outpatients	27	0.07 (0.05–0.08)	95.88, < 0.001	*P* = 0.09
	Inpatients	62	0.09 (0.07–0.10)	96.95, < 0.001	
**Species**	A	7	0.05 (0.01–0.11)	71.62, < 0.001	***P*** **< 0.001**
	B	6	0.05 (0.01–0.11)	80.03, < 0.001	
	C	8	0.29 (0.16–0.44)	88.67, < 0.001	
	D	5	0.09 (0.02–0.19)	88.08, < 0.001	
	E	2	0.05 (0.01–0.12)	NA	
	F	7	0.57 (0.41–0.72)	89.01, < 0.001	

### Distribution of species and types of HAdVs

**Fig. 3:**
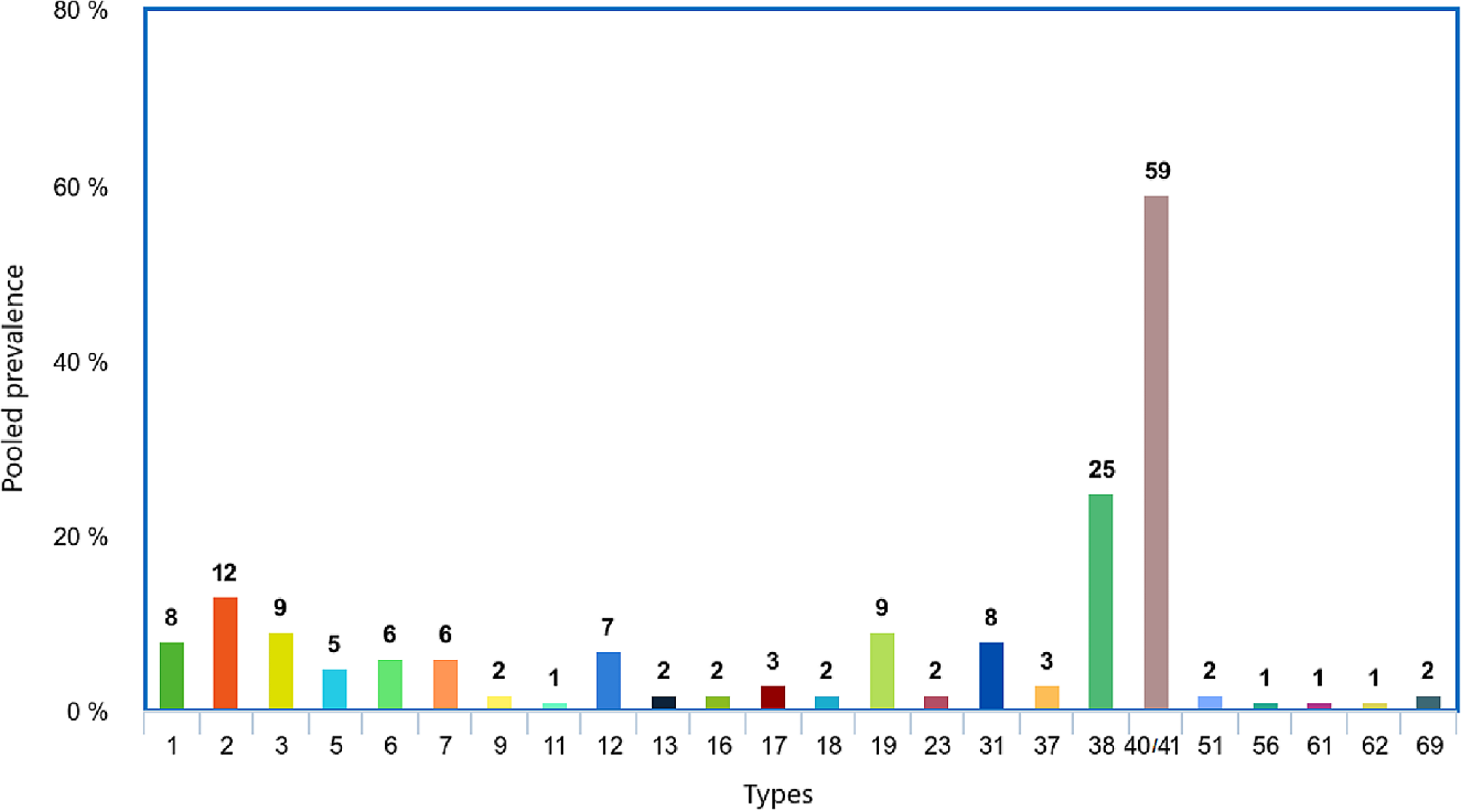
Distribution of HAdV types in children with gastroenteritis

Our results showed that the majority of HAdVs circulating in pediatric patients with gastroenteritis belonged to species F (57%; 95% CI: 41-72%) and species C (29%; 95% CI: 16-44%) (*P* < 0.001). Overall, twenty-eight types of HAdVs were detected among pediatric patients with gastroenteritis across studies. The most prevalent HAdVs observed in children with gastroenteritis were types 40/41 (59%, 95% CI: 49–68%), 38 (25%, 95% CI: 0–79%), and 2 (12%, 95% CI: 7–17%). Figure 3 shows more details on the frequency of HAdV types in children with gastroenteritis. Types 6 (20%; 95% CI: 12-28%) and 37 (5%; 95% CI: 1-14%) in Africa, types 1 (15%; 95% CI: 0-42%), 2 (29%; 95% CI: 17-43%) and 5 (12%; 95% CI: 3-28%) in Europe, types 3 (13%; 95% CI: 3-18%), 4 (1%; 95% CI: 0-2%), and 18 (4%; 95% CI: 0-10%) in Asia, and types 7 (8%; 95% CI: 2-20%), 12 (17%; 95% CI: 8-30%), 40/41 (66%; 95% CI: 17-100%) in South America were the most prevalent types in each one of the mentioned geographical areas. Africa and South America had equally the highest percentage of type 31 (Africa:12%; 95% CI: 0-33%; South America: 12%; 95% CI: 6-19% ) Analysis of other types in different continents was not possible due to lack or low number of reports.

### Prevalence of HAdV infection before and after coronavirus disease 2019 (COVID-19)

Our analysis indicated that the prevalence of HAdV among children with gastroenteritis in studies with the sampling time in 2019 and earlier was (10%, 95% CI: 9–11%) while the prevalence in studies with sampling time from 2020 and later was (13%, 95% CI: 6–21%), showing a statistically significant difference *(P* < 0.001).

### Association of HAdV infection with gastroenteritis among children

**Fig. 4:**
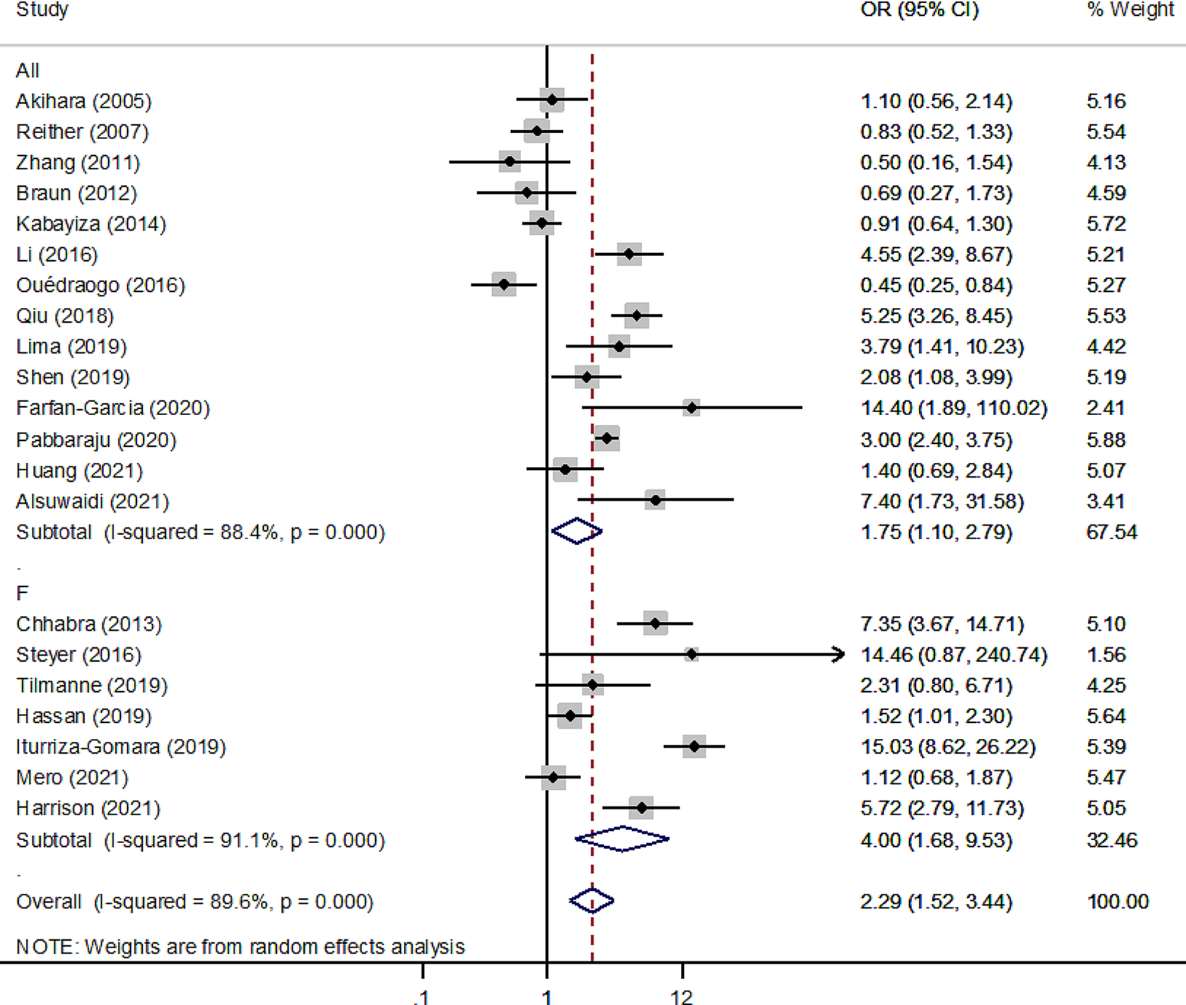
Forest plot of the association between HAdV infection and gastroenteritis risk in pediatric patients according to the random effect model in case case-control studies using universal and F primers for the detection of HAdV

The second analysis used data from case-control studies to look into the relationship between HAdV infection and the risk of gastroenteritis in children. There were 10,482 gastroenteritis patients and 7618 controls in 21 case-control studies. The results showed that the overall pooled odds ratio (OR) of the association of HAdV infection (detected by universal + species F primers) and gastroenteritis was 2.29 (95% CI: 1.52–3.44; I^2^ = 89.6%) (Fig. 4). The association was much stronger between HAdV species F (detected by species F primers) and gastroenteritis (4.0; 95% CI: 1.68–9.53; I^2^ = 91.1%) than between all types of HAdV (detected by universal primers) and gastroenteritis (1.75; 95% CI: 1.10–2.79; I^2^ = 88.4%).

**Fig. 5:**
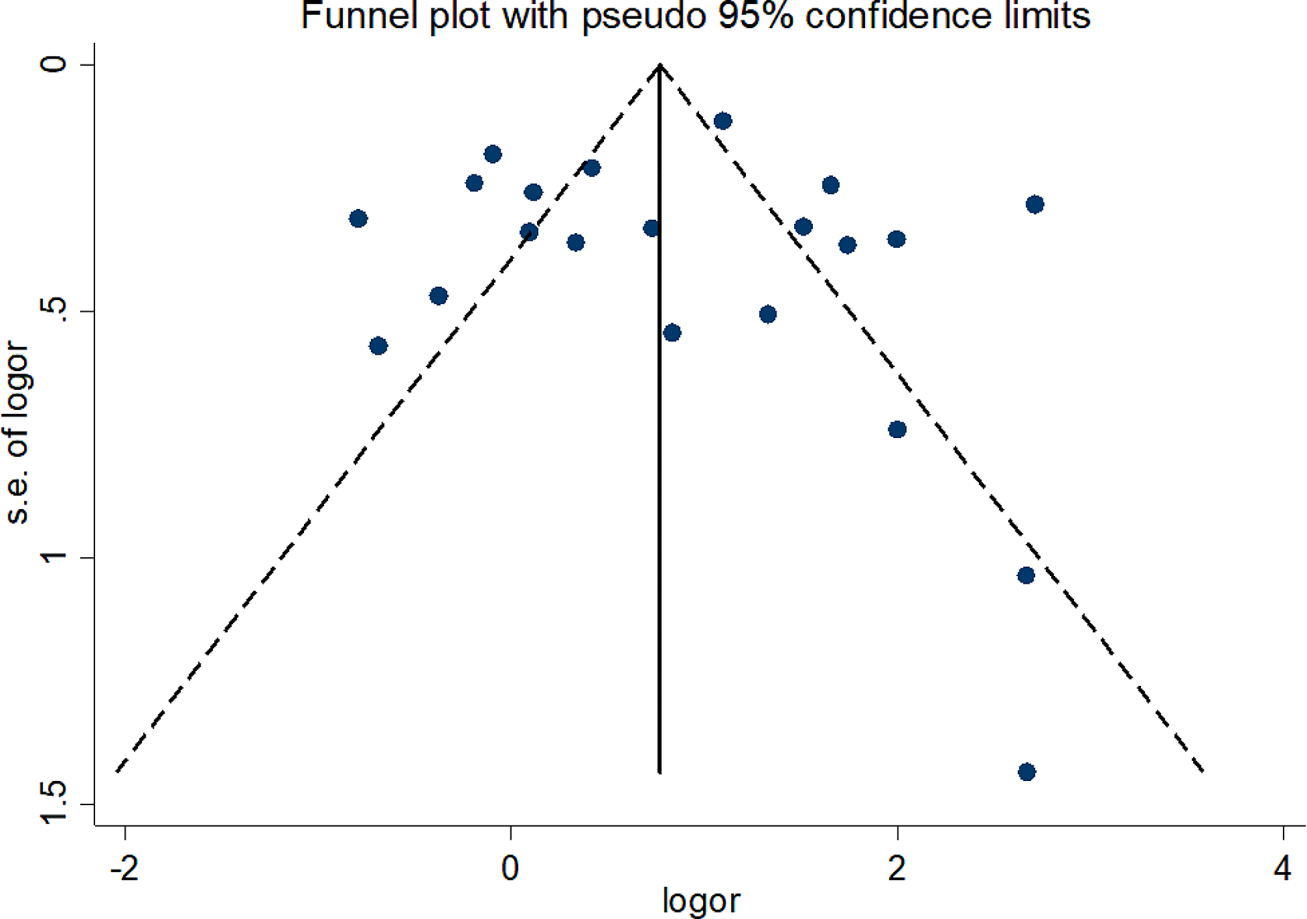
Funnel plot for assessment of publication bias

Based on the funnel plot (Fig. 5) there was no evidence of publication bias in the meta-analysis, which was statistically supported by Begg’s test (*p* = 0.55) and Egger’s test (*p* = 0.82).

### Sensitivity analysis

In a sensitivity analysis by successively removing a particular study at a time to assess the influence of every single study on pooled results, a significant positive association [range of summary ORs 2.14–2.43] between HAdV infection and gastroenteritis among children was observed consistently and did not alter the pooled results, which indicated that the meta-analysis model is robust.

## Discussion

Acute gastroenteritis is still a prominent global health threat for children, especially in developing countries. In recent years, the improvements in sanitation have led to a decrease in the prevalence of bacterial and parasitical agents in the development of acute gastroenteritis, making viruses the main causative agent of the disease. While individuals of all ages can be infected by HAdVs, children are the main targets of these viruses. To the best of our knowledge, no systematic review and meta-analysis has been performed on the prevalence of HAdVs and pediatric patients with gastroenteritis. Our results showed a high (10%) prevalence of HAdVs in children with gastroenteritis, which highlights the role of HAdVs as a main cause of gastroenteritis among children worldwide. While rotavirus was known as the main cause of pediatric gastroenteritis, the introduction of rotavirus vaccine is changing the pattern [174, 175]. We exhibited a higher prevalence of HAdVs infection in studies published after 2010, which show the increasing trend of HAdVs in the pathogenesis of pediatric gastroenteritis. Furthermore, the analysis of case-control studies indicated an association (OR: 2.28, 95% CI; 1.51–3.44) between HAdV infection and gastroenteritis in children. Therefore, in addition to respiratory infections [176], HAdVs are important pathogens in gastroenteritis among children.

Among the different regions, the highest prevalence was observed in Africa. Low micronutrition (such as vitamins and trace elements) intake as a result of malnutrition is a key factor that abates the ability of both innate and adaptive immune systems to fight pathogens [177]. Also, poor sanitation and hygiene can be a key contributor that facilitates the infection of the gastrointestinal tract by enteric viruses [178]. Other continents with high prevalence were South America and Oceania. Therefore, our results delineate an epidemiologic pattern with higher prevalence in the southern hemisphere. This can be due to malnutrition and lack of proper hygiene, which make children prone to infectious gastroenteritis by negatively affecting the immune system and exposing children to viral agents [174]. Interestingly, there was a significantly higher prevalence of HAdV in pediatric gastroenteritis cases after the initiation of the COVID-19 pandemic. It might be due to the fact that despite the effects of social distancing and mask mandate on respiratory infections, their effects on preventing viral gastroenteritis were limited and in some regions, schools were less focused on preventing gastroenteritis and therefore, school authorities could not efficiently report gastroenteritis cases, which enables the viruses to be transmitted to other children [179]. Moreover, the rise of malnutrition due to financial restrictions and lack of access to school meals for children [180] is another factor, which makes children more vulnerable to viral infection by weakening the immune system [181].

Our results did not indicate a significant difference (*P* value = 0.06) between male and female patients. This suggests that the occurrence of HAdVs-related gastroenteritis does not exhibit a gender-based pattern among children. After puberty, different compartments of the immune system are affected by sex hormones. For example, androgens including dihydrotestosterone (DHT) and testosterone can suppress immune cell activities in post-pubertal individuals [182]. However, the impacts of sex hormones are not significant in this study due to the age of the included patients. Studies on various viral infections in children resulted in different results and therefore, a specific sex is not prone to viral infections [183].

Exploring the age-specific prevalence of HAdVs in pediatric gastroenteritis showed intriguing patterns in the distribution of infections across different age groups. Notably, the prevalence of HAdVs was significantly higher (*P* < 0.001) in children younger than 5 years old, which shows the higher susceptibility of this age group. This difference aligns with the well-established notion that young children are more susceptible to viral infections. This vulnerability is primarily due to the immaturity of their immune systems, which does not provide robust protection against viral pathogens [184]. Additionally, younger children often have limited pre-existing immunity and may lack previous exposure to HAdVs, which makes them more prone to infection [185]. The increased possibility of close contact in daycare settings, preschools, and households may further contribute to a higher risk of transmission in this age group. As children advance to later childhood, their immune systems become more mature [184] and exposure in early life fosters a natural immunity to pathogens [174, 184]. Behavioral changes such as improved hygiene practices may also help the reduction of the risk of diarrhea [186]. These findings highlight the importance of monitoring HAdV transmission and infection in childcare and healthcare settings to avoid future outbreaks.

More than half of typed HAdVs in our study belonged to species F, which consisted of types 40/41 that are known for their role in gastroenteritis [11]. In addition, no significant difference (*P* = 0.06) was observed between universal primers and those that were designed to only detect species F, which shows the remarkable prevalence of this species in HAdVs-related gastroenteritis cases. Interestingly, we observed that species C, which is known to cause respiratory symptoms [11], is the second most prevalent species in pediatric gastroenteritis cases. This underscores the clinical importance of species C as a causative agent of respiratory and gastrointestinal infections among children. These data are good indicators of the most prevalent species and can be used to design effective vaccines.

In the context of clinical settings, the pooled prevalence for inpatients was found to be slightly higher than the prevalence among outpatients. While this difference did not reach statistical significance (*P* = 0.09), the trend suggests a potential association between HAdV infections and increased disease severity necessitating hospitalization. Noteworthy, the lack of statistical significance may stem from various factors, including heterogeneity in study populations, variations in healthcare practices, and potential underreporting in outpatient settings. Further research into the specific factors contributing to the observed prevalence differences between inpatients and outpatients can provide valuable insights into the clinical implications of HAdVs-associated gastroenteritis.

This systematic review and meta-analysis faced some limitations. There were no studies from some countries in various geographic regions such as Africa and Europe. We recommend researchers to conduct epidemiologic studies in those countries with no previous reports to gain a comprehensive insight into the HAdV epidemiology in pediatric gastroenteritis. Some reports did not mention the characteristics of isolated viruses including species and genotypes. Finally, in the context of systematic review and meta-analysis studies, publication bias and study heterogeneity are inevitable limitations.

## Conclusion

This systematic review and meta-analysis highlights HAdVs as significant and increasing causes of pediatric gastroenteritis globally, particularly affecting children under 5 years old. The prevalence is considerably high in Africa, with also remarkable rates in South America and Oceania, which shows a southern hemisphere predominance possibly linked to factors such as malnutrition and poor sanitation. Furthermore, the absence of a gender-based pattern suggests equal susceptibility among male and female pediatric patients. Variations in diagnostic approaches indicate the importance of choosing sensitive tests such as Nested PCR. The dominance of species F adenoviruses, genotypes 40/41, shows potential targets for vaccine development. A higher prevalence among inpatients can be indicative of the potential of HAdVs to cause severe gastrointestinal symptoms. These results suggest future epidemiologic investigations, particularly in underrepresented regions to address existing gaps in HAdVs epidemiology in pediatric gastroenteritis.

### Electronic supplementary material

Below is the link to the electronic supplementary material.

Supplementary Material 1

## Data Availability

The data that support the findings of this study are available from the corresponding author upon reasonable request.
