# Supplementary material for: Human adenoviruses in children with gastroenteritis: a systematic review and meta-analysis
Source: BMC Infect Dis. 2024 May 9;24:478. doi: 10.1186/s12879-024-09386-x (PMC11084101; doi:10.1186/s12879-024-09386-x)
Supplement: Supplementary file 1 — Supplementary Material 1 [file 12879_2024_9386_MOESM1_ESM.docx]

**Table S1.** The full details of the search strategy for each database.

| **Database** | **Search terms** |
| --- | --- |
| PubMed | ((Adenovirus [Title/Abstract] OR Adenoviruses [Title/Abstract] OR HAdV [Title/Abstract] OR “Human adenovirus” [Title/Abstract] OR “Human adenoviruses” [Title/Abstract]) AND (gastroenteritis [Title/Abstract] OR Diarrhea [Title/Abstract]) AND (Pediatric [Title/Abstract] OR Pediatrics [Title/Abstract] OR Child [Title/Abstract] OR Children [Title/Abstract] OR Infant [Title/Abstract] OR Newborn [Title/Abstract] OR Neonate [Title/Abstract] OR Adolescent [Title/Abstract])) |
| **Scopus** | TITLE-ABS-KEY ( (Adenovirus OR Adenoviruses OR HAdV OR " Human adenovirus" OR " Human adenoviruses") AND ( gastroenteritis OR diarrhea ) AND ( pediatric OR pediatrics OR child OR children OR infant OR newborn OR neonate OR adolescent ) ) |
| **Web of science** | (TS=(( Adenovirus OR Adenoviruses OR HAdV OR " Human adenovirus" OR " Human adenoviruses") AND (gastroenteritis OR diarrhea ) AND ( pediatric OR pediatrics OR child OR children OR infant OR newborn OR neonate OR adolescent ))) AND LANGUAGE: (English) AND DOCUMENT TYPES: (Article) |
